# Draft Genome Sequence of the Microcystin-Degrading Bacterium *Novosphingobium* sp. Strain MD-1

**DOI:** 10.1128/MRA.01413-19

**Published:** 2020-03-19

**Authors:** Kunihiro Okano, Kazuya Shimizu, Takeshi Saito, Hideaki Maseda, Motoo Utsumi, Tomoaki Itayama, Norio Sugiura

**Affiliations:** a Department of Biological Environment, Faculty of Bioresource Sciences, Akita Prefectural University, Akita, Japan; b Faculty of Life and Environmental Sciences, University of Tsukuba, Ibaraki, Japan; c R&D Biological Science Research, Kao Corporation, Wakayama, Japan; d National Institute of Advanced Industrial Science and Technology, Osaka, Japan; e Microbiology Research Center for Sustainability, University of Tsukuba, Ibaraki, Japan; f Graduate School of Engineering, Nagasaki University, Nagasaki, Japan; g Malaysia-Japan International Institute of Technology, Universiti Technologi Malaysia, Kuala Lumpur, Malaysia; University of Southern California

## Abstract

This report describes the whole-genome sequence of a microcystin-degrading bacterium, *Novosphingobium* sp. strain MD-1, isolated from a lake in Japan. The *Novosphingobium* sp. strain MD-1 genome had a total length of 4,617,766 bp. Moreover, strain MD-1 showed a conserved microcystin-degrading gene cluster (*mlrA* to *mlrF*), similar to *Sphingopyxis* sp. strain C-1.

## ANNOUNCEMENT

Harmful algal blooming cyanobacteria such as *Microcystis* spp. produce microcystin, which is a potent hepatotoxin. Microcystin is very stable (1, 2) but can be degraded by specific enzymes (MlrA, MlrB, MlrC, and MlrD) in microcystin-degrading bacteria (3–6). Lakes and reservoirs that have harmful algal blooms generally have highly alkaline pH (7), and alkaline conditions provide the optimal pH for most microcystin-degrading bacteria (8). However, the microcystin-degrading bacterium *Novosphingobium* sp. strain MD-1 (formerly classified as *Sphingomonas* sp. strain MD-1) prefers a neutral pH (9, 10). Therefore, the whole gene structure may differ greatly from that of other microcystin-degrading bacteria. Here, we report the whole-genome sequence of *Novosphingobium* sp. strain MD-1.

*Novosphingobium* sp. strain MD-1 was isolated from Lake Kasumigaura in 1999 (9). *Novosphingobium* sp. strain MD-1 has been maintained in a glycerol stock at −80°C. Culturing was conducted using 1:5 peptone-yeast extract (PY) at 28°C, and then total DNA was extracted in the late logarithmic growth phase. Genomic DNA was prepared as a paired-end library (350 bp) with the TruSeq Nano DNA low-throughput (LT) sample preparation kit (Illumina, San Diego, CA, USA) and as a mated-pair library (8 to 10 kb) with a Nextera mate pair sample preparation kit (Illumina). Whole-genome sequencing was carried out using an Illumina HiSeq 2500 system with a paired-end library and a mated-pair library. A total of 22,852,894 reads, averaging 99 bp long, were obtained, for a total of 2,262,436,506 bases of the sequence. The final coverage (depth) of the filtered genome sequence was 146×. All reads were assembled *de novo* using Velvet v1.2.08 (11). Gaps between the resultant 292 contigs were closed using Platanus v1.2.1 (12). The whole genome was annotated using the RAST server (http://rast.nmpdr.org/rast.cgi), which predicted protein-coding sequences (CDSs). Default parameters were used for all software.

The *Novosphingobium* sp. strain MD-1 genome consisted of 34 contigs, with an *N*_50_ value of 406,083 bp, and had a total length of 4,617,766 bp. The draft genome had a G+C content of 65.80%, with 4,178 CDSs and 60 RNA-coding genes (i.e., 3 sets of rRNA genes and 51 tRNA genes). The annotation revealed that 3,060 CDSs exhibited homology to genes with known functions; the remaining 1,118 CDSs encoded hypothetical proteins with unknown functions.

Okano et al. reported that microcystin is degraded by six enzymes in *Sphingopyxis* sp. strain C-1, namely, MlrA, MlrB, MlrC, MlrD, MlrE, and MlrF (13). The *mlrF*, *mlrE*, *mlrB*, *mlrD*, *mlrA*, and *mlrC* genes of *Novosphingobium* sp. strain MD-1 were designated with locus tags NMD1_03009 to NMD1_03013, respectively (positions 426855 to 435216), in the genome (GenBank accession number BBXA01000030). MlrA, MlrB, MlrC, MlrD, MlrE, and MlrF had 93.3, 95.7, 93.7, 95.3, 96.8, and 90.7% identities, respectively, to those of *Sphingopyxis* sp. strain C-1 on an amino acid basis, using the Align Sequences Protein BLAST. Therefore, it was suggested that the six genes participated in microcystin degradation independent of the suitable pH for growth.

### Data availability.

This whole-genome shotgun project was deposited in DDBJ/EMBL/GenBank under accession number BBXA00000000. Raw HiSeq data were deposited in the DDBJ Sequence Read Archive under accession number DRA009321.
